# Performance Comparison of the Digital Neuromorphic Hardware SpiNNaker and the Neural Network Simulation Software NEST for a Full-Scale Cortical Microcircuit Model

**DOI:** 10.3389/fnins.2018.00291

**Published:** 2018-05-23

**Authors:** Sacha J. van Albada, Andrew G. Rowley, Johanna Senk, Michael Hopkins, Maximilian Schmidt, Alan B. Stokes, David R. Lester, Markus Diesmann, Steve B. Furber

**Affiliations:** ^1^Institute of Neuroscience and Medicine (INM-6), Institute for Advanced Simulation (IAS-6), JARA Institute Brain Structure-Function Relationships (INM-10), Jülich Research Centre Jülich, Germany; ^2^Advanced Processor Technologies Group, School of Computer Science, University of Manchester, Manchester, United Kingdom; ^3^Laboratory for Neural Circuit Theory, RIKEN Brain Science Institute, Wako, Japan; ^4^Department of Physics, Faculty 1, RWTH Aachen University, Aachen, Germany; ^5^Department of Psychiatry, Psychotherapy and Psychosomatics, Medical Faculty, RWTH Aachen University, Aachen, Germany

**Keywords:** neuromorphic computing, high-performance computing, parallel computing, accuracy of simulation, energy to solution, benchmarking, strong scaling, computational neuroscience

## Abstract

The digital neuromorphic hardware SpiNNaker has been developed with the aim of enabling large-scale neural network simulations in real time and with low power consumption. Real-time performance is achieved with 1 ms integration time steps, and thus applies to neural networks for which faster time scales of the dynamics can be neglected. By slowing down the simulation, shorter integration time steps and hence faster time scales, which are often biologically relevant, can be incorporated. We here describe the first full-scale simulations of a cortical microcircuit with biological time scales on SpiNNaker. Since about half the synapses onto the neurons arise within the microcircuit, larger cortical circuits have only moderately more synapses per neuron. Therefore, the full-scale microcircuit paves the way for simulating cortical circuits of arbitrary size. With approximately 80, 000 neurons and 0.3 billion synapses, this model is the largest simulated on SpiNNaker to date. The scale-up is enabled by recent developments in the SpiNNaker software stack that allow simulations to be spread across multiple boards. Comparison with simulations using the NEST software on a high-performance cluster shows that both simulators can reach a similar accuracy, despite the fixed-point arithmetic of SpiNNaker, demonstrating the usability of SpiNNaker for computational neuroscience applications with biological time scales and large network size. The runtime and power consumption are also assessed for both simulators on the example of the cortical microcircuit model. To obtain an accuracy similar to that of NEST with 0.1 ms time steps, SpiNNaker requires a slowdown factor of around 20 compared to real time. The runtime for NEST saturates around 3 times real time using hybrid parallelization with MPI and multi-threading. However, achieving this runtime comes at the cost of increased power and energy consumption. The lowest total energy consumption for NEST is reached at around 144 parallel threads and 4.6 times slowdown. At this setting, NEST and SpiNNaker have a comparable energy consumption per synaptic event. Our results widen the application domain of SpiNNaker and help guide its development, showing that further optimizations such as synapse-centric network representation are necessary to enable real-time simulation of large biological neural networks.

## 1. Introduction

Tools for simulating neural networks fall into two categories: simulation software and neuromorphic hardware. The available features, the speed at which the simulation engine arrives at the solution, and the power consumption differ between tools, but the tools are rarely systematically compared. To find out where we stand and to provide guidance for future research, we need to learn how to port network models discussed in the current literature from conventional software implementations to neuromorphic hardware and how to quantitatively compare performance.

The distinction between simulation software and neuromorphic hardware is not clear-cut. Next to the hardware, a neuromorphic system readily usable by neuroscientists requires a multi-level software stack engaging in tasks from the interpretation of a domain-specific model description language to the mapping of the neural network to the topology of the neuromorphic hardware. Reversely, simulation software profits from computer hardware adapted to the microscopic parallelism of neural networks with many computational cores and a tight integration of processing hardware and memory. For the purpose of the present study we refer to simulation software as a system that runs on conventional high-performance computing hardware without dedicated neuromorphic hardware.

The time as well as the energy required to arrive at the solution are becoming relevant as neuroscientists turn to supercomputers to simulate brain-scale neural networks at cellular resolution. Today's supercomputers require tens of minutes to simulate one second of biological time and consume megawatts of power (Kunkel et al., 2014; Jordan et al., 2018). This means that any studies on processes like plasticity, learning, and development exhibited over hours and days of biological time are outside our reach.

Although this is sometimes forgotten, not only speed and power consumption but also the accuracy of the simulation results is of importance: a highly inaccurate solution can be obtained arbitrarily fast. In other words, a statement on the wall clock time required to arrive at the solution is meaningless without a statement on the achieved accuracy. Like runtime, energy consumption depends on the level of simulation accuracy. Low energy consumption is emphasized in the development of neuromorphic hardware, but accuracy is generally not explicitly taken into account when characterizing energy consumption. How one quantifies accuracy should be determined in the light of the trade-off between the combination of precision and flexibility on the one hand and the combination of speed and energy efficiency on the other hand which is the main idea behind dedicated hardware. If a dedicated hardware trades precision for speed and energy efficiency, for instance by having noisy components or not delivering every single spike, this is acceptable if the given precision still yields the desired network behavior. The relevant issue is then not whether but how to assess accuracy, that is, defining how the network should behave.

Here, we consider as a use case the digital neuromorphic hardware SpiNNaker (Furber et al., 2013) and the neural network simulation software NEST (Gewaltig and Diesmann, 2007), both in use by the neuroscientific community and supporting the simulator-independent description language PyNN (Davison et al., 2008). Both NEST and SpiNNaker are designed to enable the simulation of large neural network models. SpiNNaker enhances its efficiency through asynchronous update where spikes are processed as they come in and are dropped if the receiving process is busy over several delivery cycles. It is especially suited to robotic applications enabling the simulation to operate in real-time, but since it is general-purpose, in principle any type of neural network model can be simulated, including biological and artificial neural networks. In the context of the European Human Brain Project (HBP) a large system is under construction at the University of Manchester targeting brain-scale simulations. The networks in question may have static synapses or include plasticity. For simplicity, and since there is a close relationship between simulator performance with and without synaptic plasticity (e.g., Knight and Furber, 2016), we here focus on a non-plastic network: a spiking cortical microcircuit model (Potjans and Diesmann, 2014).

The microcircuit is regarded as unit cell of cortex repeated to cover larger areas of cortical surface and different cortical areas. The model represents the full density of connectivity in 1 mm^2^ of the cortical sheet by about 80, 000 leaky integrate-and-fire (LIF) model neurons and 0.3 billion synapses. This is the smallest network size where a realistic number of synapses and a realistic connection probability are simultaneously achieved. The capability to simulate this model constitutes a breakthrough as larger cortical models are necessarily less densely connected, with only a limited increase in the number of synapses per neuron for increased model size. Consequently, from this network size on, the computer memory required to store the synaptic parameters grows close to linearly with network size (Lansner and Diesmann, 2012). Further, a simulation technology can be devised such that the memory consumption of a compute node is independent of the total number of neurons in the network (Jordan et al., 2018). This renders the total memory consumption approximately directly proportional to the number of neuronal and synaptic elements in the model. The model already serves as a building block for a number of further studies and larger networks (Wagatsuma et al., 2011; Cain et al., 2016; Hagen et al., 2016; Schmidt et al., 2016; Schwalger et al., 2017), and a first comparison of the simulation results of NEST and SpiNNaker for this model has served as a test case for a workflow implementation on the collaboration platform of the Human Brain Project (Senk et al., 2017). The original implementation uses NEST, which can also handle much larger networks with trillions of synapses (RIKEN BSI, 2013; Kunkel et al., 2014; Forschungszentrum Jülich, 2018; Jordan et al., 2018) under the increased memory consumption and run time costs indicated above. The previously largest simulations on SpiNNaker comprised about 50 million (Sharp et al., 2014; Knight et al., 2016) and 86 million synapses (Stromatias et al., 2013). Thus, the present study describes the largest simulation on SpiNNaker to date, and also the first to implement the connectivity at full biological density.

SpiNNaker achieves real-time performance for an integration time step of 1 ms, which is suited to networks with dynamics on time scales sufficiently greater than 1 ms. While a resolution of 1 ms generally suffices for today's applications in robotics and artificial neural networks, a time step of 0.1 ms is typical for neuroscience applications due to the neurobiological time scales and the need to avoid artifacts of global synchronization (Morrison et al., 2007b). The model of Potjans and Diesmann (2014) has synaptic time constants of 0.5 ms, and therefore requires integration time steps smaller than this. The current software controlling SpiNNaker enables using small time steps by slowing down the simulation. In the present work, we show how this feature in combination with further improvements of the software stack allows the cortical microcircuit model to be accurately integrated. This result demonstrates the usability of SpiNNaker for large-scale neural network simulations with biologically realistic time scales.

To assess accuracy, we compare simulation results with a reference solution obtained with an alternative solver (Morrison et al., 2007b; Hanuschkin et al., 2010) available in the NEST simulation code where spikes are not restricted to the grid spanned by the computation step size. The spike times from the cortical microcircuit model obtained with different simulation engines can only be compared in a statistical sense. Therefore we also look at single-neuron accuracy (Henker et al., 2012). Here, we consider both the 0.1 ms time step used in the microcircuit simulations, and 1 ms, the original design specification of SpiNNaker, and further, we investigate different spike rates to vary the relative contributions of subthreshold and spiking activity. This is relevant because NEST integrates the subthreshold dynamics exactly (Rotter and Diesmann, 1999), whereas SpiNNaker uses exponential integration (MacGregor, 1987), in which the synaptic currents are treated as piecewise constant. For the microcircuit model, we characterize accuracy based on distributions of spike rates, spike train irregularity, and correlations. Spike rates are chosen as a first-order measure of neural activity, and correlations together with spike train irregularity are relevant because cortical activity is known to be asynchronous irregular (van Vreeswijk and Sompolinsky, 1998); mesoscopic measures of brain activity like the local field potential (LFP) primarily reflect correlations in the microscopic dynamics (Hagen et al., 2016); and correlations in spiking activity drive further aspects of network dynamics like spike-timing-dependent plasticity (STDP; Morrison et al., 2007a) underlying system-level learning. The three aforementioned measures of spiking activity are also the focus in the work of Potjans and Diesmann (2014).

Previous work has evaluated the energy consumption of various types of processors (Hasler and Marr, 2013) including SpiNNaker (Sharp et al., 2012; Stromatias et al., 2013) in relation to the number of operations performed. Here, we take a different approach, comparing the energy consumption of two simulation engines under the condition of comparable accuracy. This accuracy depends not only on the number of operations of a given precision, but also on the algorithms employed. For comparison with previous results (Sharp et al., 2012; Stromatias et al., 2013), we further derive the energy consumed per synaptic event.

In the following, we compare the accuracy of single-neuron LIF simulations between NEST and SpiNNaker, describe the adjustments made to SpiNNaker to enable the cortical microcircuit model to be implemented, and compare both simulators in terms of accuracy, runtime, and energy consumption. We also discuss the sources of differences in simulation results and performance between NEST and SpiNNaker. Thus, our study enables neuromorphic engineers to learn more about the internal workings of SpiNNaker and the implications for performance, and brings SpiNNaker closer to being a tool of choice for computational neuroscience use cases with large network size and short biological time scales.

Preliminary results have been presented in abstract form (van Albada et al., 2016, 2017).

## 2. Methods

### 2.1. The leaky integrate-and-fire neuron model

The cortical microcircuit model uses leaky integrate-and-fire (LIF) model neurons with synaptic currents modeled as jumps followed by an exponential decay. The subthreshold dynamics of each neuron is given by

$$\begin{array}{l} \tau_{\text{m}}\frac{\text{d}V_{i}}{\text{d}t}=-\left(V_{i}-E_{\text{L}}\right)+R_{\text{m}}I_{i}\left(t\right),\\ \tau_{\text{s}}\frac{\text{d}I_{i}}{\text{d}t}=-I_{i}+\tau_{\text{s}}\underset{j}{\sum }J_{ij}s_{j}\left(t-d_{j}\right), \end{array}$$

where τ_m_ and τ_s_ are membrane and synaptic time constants, *E*_L_ is the leak or resting potential, *R*_m_ is the membrane resistance, *V*_*i*_ is the membrane potential of neuron *i*, *I*_*i*_ is the total synaptic current onto the neuron, *J*_*ij*_ is the jump in the synaptic current due to a single spike from neuron *j*, $s_{j}=\underset{k}{\sum }\delta \left(t-t_{k}^{j}\right)$ are the incoming spike trains, and *d*_*j*_ is the transmission delay. When *V*_*i*_ reaches a threshold θ, a spike is emitted, and the membrane potential is clamped to a level *V*_r_ for a refractory period τ_ref_. Table 1 lists the single-neuron parameters.

**Table 1:** Parameters of the leaky integrate-and-fire model neurons used in the simulations.

Membrane time constant	τ_m_	10 ms
Synaptic time constant	τ_s_	0.5 ms
Refractory period	τ_ref_	2 ms
Membrane resistance	*R*_m_	40 MΩ
Leak potential	*E*_L_	−65 mV
Threshold	θ	−50 mV
Reset potential	*V*_r_	−65 mV

NEST integrates this model using exact integration (Rotter and Diesmann, 1999), so that the subthreshold dynamics precisely follows the analytical solution. The spikes can either be constrained to the time grid or interpolated between grid points to yield precise spike times (Morrison et al., 2007b; Hanuschkin et al., 2010). In the present study, we consider both options, the latter providing a reference solution. For reasons of modularity, SpiNNaker separates the neuron and synapse dynamics and uses exponential integration (MacGregor, 1987; reviewed in Rotter and Diesmann, 1999), in which the input current to the membrane potential equation is treated as piecewise constant. The synaptic currents are decayed over one time step before being added to the input, to ensure that the total charge transferred per synaptic event is *Jτ*_s_, as in the exact solution.

### 2.2. Single-neuron tests

Simple systems such as single LIF model neurons allow a deterministic assessment of simulation accuracy (Henker et al., 2012). We assess the accuracy of NEST and SpiNNaker by comparing with precise solutions the subthreshold and spiking dynamics of single LIF model neurons receiving excitatory Poisson input with synaptic strength 87.8 pA, equal to the mean synaptic strength for the excitatory connections between most populations in the network model. The study considers both integration time steps of 0.1 ms to match the network simulations, and 1 ms, matching the primary design specification of SpiNNaker. Two input rates are investigated: 8, 000 spikes/s (giving an output rate of around 17 spikes/s) and 10, 000 spikes/s (giving an output rate of around 47 spikes/s), to study different proportions of subthreshold activity and spiking. As in the network simulations, the input spikes are constrained to the time grid. The simulations with the lower input rate are run for 16 s and those with the higher input rate for 4 s biological time to yield comparable total numbers of spikes for the low-rate and high-rate simulations, and a total of 10 simulations with different random seeds are performed for each setting. The other neuron parameters are as in the network model.

We characterize the accuracy of the single-neuron simulations in four ways, in each case comparing with NEST simulations with precise spike timing: (1) cross-correlation histograms of spike times with bin width equal to half the integration time step; (2) Pearson correlation coefficients between membrane potential traces recorded at the integration time steps; (3) the average percentage lead or lag in spike times; and (4) the root mean square error (RMSE) of the spike times after correcting for the average lead or lag. To determine the accumulated percentage lead or lag in spike times, we first find *N* = min(*N*_spikes_[precise], *N*_spikes_[discrete]), where discrete refers to simulations with SpiNNaker or with NEST with spikes constrained to the grid and *N*_spikes_ is the total number of spikes in the respective simulation. The accumulated fractional lead or lag in spike times is then computed as [*t*_discrete_(*N*)−*t*_precise_(*N*)]/*t*_discrete_(*N*), where *t*_precise_(*N*) and *t*_discrete_(*N*) refer to the time of the *N*th spike in the respective simulation. The correction for the accumulated lead or lag before determining the RMSE is performed to obtain a measure of the variability of the spike times independent of overall rate differences between simulation methods. It consists of warping the spike times in the discrete simulation by the factor *t*_precise_(*N*)/*t*_discrete_(*N*) such that the last spike times considered coincide. Denoting the resulting spike times as $t_{\text{discrete}}^{*}$, the RMSE is then determined as $\sqrt{\overset{N}{\underset{i=1}{\sum }}{\left(t_{\text{discrete}}^{*}\left(i\right)-t_{\text{precise}}\left(i\right)\right)}^{2}/N}$.

### 2.3. Cortical microcircuit model

**Figure 1:**
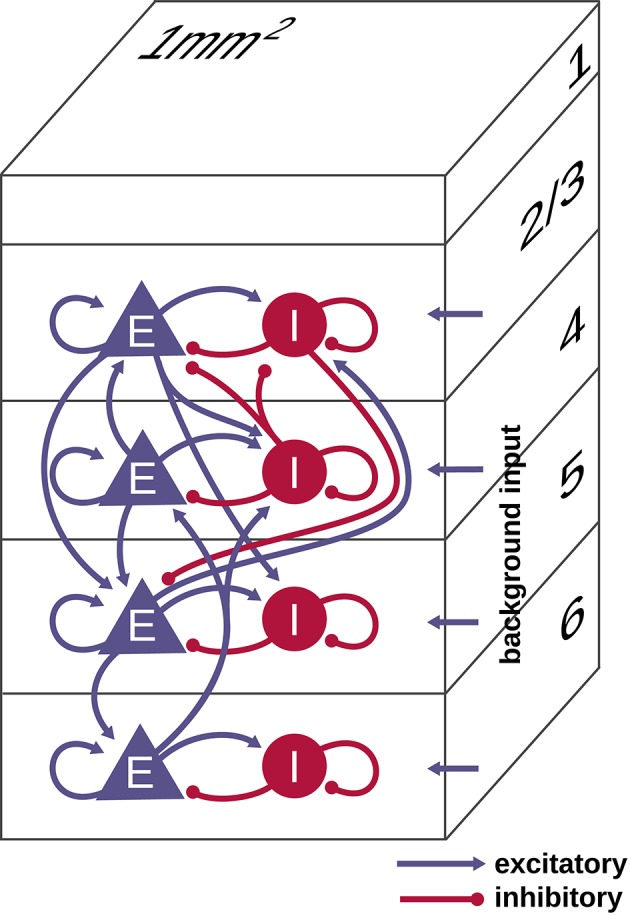
Schematic illustration of the microcircuit model of early sensory cortex. The model represents 1 mm^2^ of cortex with the full density of neurons and synapses, for a total of 77, 169 neurons and about 3 × 10^8^ synapses. Each of the layers 2/3, 4, 5, and 6 contains an excitatory (E) and inhibitory (I) population of leaky integrate-and-fire model neurons. All neurons receive an external Poisson drive representing inputs from the rest of the brain. Figure adapted from Potjans and Diesmann (2014) with permission.

The model, taken from Potjans and Diesmann (2014), represents the neurons under 1 mm^2^ of surface of generic early sensory cortex, organized into layers 2/3, 4, 5, and 6 (see Figure 1). It comprises 77, 169 neurons connected via approximately 3 × 10^8^ synapses, with population-specific connection probabilities based on an extensive survey of the anatomical and physiological literature. The connectivity is otherwise random, drawing both source and target neurons with replacement. Each layer contains one excitatory and one inhibitory population of LIF model neurons. We denote the eight populations by 2/3E, 2/3I, 4E, 4I, 5E, 5I, 6E, and 6I. The synaptic strengths *J*_*ij*_ are normally distributed with mean ± standard deviation of 351.2 ± 35.21 pA for inhibitory source neurons and 87.8 ± 8.78 pA for excitatory source neurons except for connections from 4E to 2/3E, which have weights 175.6 ± 8.78 pA. Transmission delays are normally distributed with mean ± standard deviation of 1.5 ± 0.75 ms for excitatory source neurons and 0.75 ± 0.375 ms for inhibitory source neurons, truncated at the simulation time step. All neurons receive independent Poisson inputs with population-specific rates reflecting connections from adjacent cortex, other cortical areas, and subcortical regions. For further details we refer to Potjans and Diesmann (2014).

We run the simulations over 10 s of biological time with a time step of 0.1 ms, the original time step used for simulating the model and one of the time steps for which SpiNNaker is designed. In one set of simulations, the Poisson input of the original model is replaced by a DC drive corresponding to its mean current. The second set of simulations uses Poisson input, drawn independently for each simulation. A 1 s transient is discarded before analysis. The accuracy of the network simulations is assessed by statistical comparisons with NEST simulations with precise spike timing, which avoid synchronization artifacts (Morrison et al., 2007b; Hanuschkin et al., 2010). Specifically, we compute the Kullback-Leibler divergence *D*_KL_ between three sets of smoothed histograms for each neural population; (1) single-neuron firing rates averaged over the simulation duration; (2) single-neuron coefficients of variation of interspike intervals (CV ISI); (3) Pearson correlation coefficients between spike trains binned at 2 ms (corresponding to the refractory time) from all disjoint neuron pairs within a subpopulation of 200 neurons, which provides a trade-off between statistical precision and computation time. For each neural population and dynamic variable, the histogram bin sizes are determined using the Freedman-Diaconis rule (Freedman and Diaconis, 1981) on the histograms for NEST with precise spike timing, $\text{binsize}=2\frac{\text{IQR}\left(x\right)}{\sqrt[{3}]{n}}$ with IQR the interquartile range and *n* the number of observations. For each population and variable, we determine *D*_KL_(*P*||*Q*) where *Q* represents the grid-based NEST or SpiNNaker data, and *P* represents the data from NEST with precise spike timing. The histograms are first smoothed via Gaussian kernel density estimation using the scipy.stats.gaussian_kde function with bandwidth 0.3 s^−1^ for the rates, 0.04 for the CV ISIs, and 0.002 for the correlations. To avoid excessive contributions of low-probability bins due to division by vanishingly small numbers, bins where the normalized histograms have values smaller than 10^−15^ are ignored. We do not perform significance tests on the results, because we know the ground truth: the simulation methods differ. The comparisons between the simulation methods therefore focus on the sizes of the differences between the outputs.

### 2.4. Implementation

The network model was originally implemented in the native simulation language interpreter (SLI) of NEST. To allow execution also on SpiNNaker and to unify the model description across back ends, we developed an alternative implementation in the simulator-independent language PyNN (version 0.7; Davison et al., 2008). On SpiNNaker, this works in conjunction with the sPyNNaker software (Rowley et al., 2015).

The NEST (version 2.8; Eppler et al., 2015) simulations are performed on a high-performance computing (HPC) cluster with 32 compute nodes. Each node is equipped with 2 Intel Xeon E5-2680v3 processors with a clock rate of 2.5 GHz, 128 GB RAM, 240 GB SSD local storage, and InfiniBand QDR (40 Gb/s). With 12 cores per processor and 2 hardware threads per core, the maximum number of threads per node using hyperthreading is 48. The cores can reduce and increase the clock rate (up to 3.3 GHz) in steps, depending on demand and thermal and power limits. Two Rack Power Distribution Units (PDUs) from Raritan (PX3-5530V) are used for power measurements. The HPC cluster uses the operating system CentOS 7.1 with Linux kernel 3.10.0. For memory allocation, we use jemalloc 4.1.0 in this study (see Ippen et al., 2017, for an analysis of memory allocation in multi-threaded simulations).

The SpiNNaker simulations are performed using the 4.0.0 release of the software stack. The microcircuit model is simulated on a machine consisting of 6 SpiNN-5 SpiNNaker boards, using a total of 217 chips and 1934 ARM9 cores. Each board consists of 48 chips and each chip of 18 cores, resulting in a total of 288 chips and 5174 cores available for use. Of these, two cores are used on each chip for loading, retrieving results and simulation control. Of the remaining cores, only 1934 are used, as this is all that is required to simulate the number of neurons in the network with 80 neurons on each of the neuron cores. Cores are also used for simulating delays of greater than 16 time steps using a “delay extension” implementation, and for simulating the Poisson input noise. Each of these cores also simulates 80 units per core, i.e., 80 sources in the case of the Poisson sources, and the extra delay for 80 neurons in the case of the delay extensions.

The given number of neurons per core was chosen as this is the smallest number of neurons that can be simulated on each core for this particular network, whilst still being able to allocate routing keys to the neurons and having the SpiNNaker routing tables fit within the hardware constraints of the machine with the current software implementation. The routing tables grow as the problem is distributed across more chips on the machine, as this requires additional paths to be made to allow the cores to communicate. The fact that the number of neurons per core cannot be reduced further also restricts the maximum speed with which the network can be simulated given the network traffic rates of the microcircuit; we find that we need to slow the simulation down by a factor of 20 from real time to maintain a 0.1 ms time step and be able to process all the spikes without overrunning the time allocated for each step. The simulation could otherwise run faster by having fewer neurons per core, and so less work to do on each core.

Whereas NEST represents parameters and dynamic variables as double-precision floating-point numbers, SpiNNaker uses the ISO draft s16.15 fixed-point arithmetic type.

All analyses are carried out with Python 2.7.9, using the Elephant package (version 0.2.1; Yegenoglu et al., 2016) for computing spike train statistics.

### 2.5. Performance benchmarks

We compare the power efficiency and runtime of the microcircuit simulations with DC input between NEST and SpiNNaker. For NEST, the strong-scaling efficiency of the simulation is assessed on the HPC cluster. In line with NEST's hybrid parallelization strategy, we use one MPI process per compute node and OpenMP-based multi-threading within each process. Since NEST internally treats threads like MPI processes, they are also referred to as “virtual processes,” and the total number of virtual processes *vp* equals the number of MPI processes times the number of threads per MPI process (Plesser et al., 2007; Kunkel et al., 2014). For the benchmark simulations, *vp* is increased from one single thread on one compute node up to the saturation of the full cluster. One compute node is first filled with one thread per core, first on one and then on the second processor, before threads are also assigned to the second available hardware thread of each core (hyperthreading). During the benchmark simulations, the power consumption of the compute nodes under load is measured with the PDUs. The active power is read approximately once per second remotely from the PDUs using the Simple Network Management Protocol (SNMP). To account for additional contributions to the overall power consumption, we furthermore estimate the usage of service nodes and switches (2 Ethernet and 1 InfiniBand) based on PDU measurements and data sheets.

Timestamps in the simulation scripts allow the identification of different execution phases, such as “network construction” and “state propagation,” and to relate them directly to the temporally resolved power measurements. For the phase during which the dynamical state of the neural network is propagated, we compute the average power consumption, the energy consumption and the energy per synaptic event. The energy consumption is obtained by integrating the measured power. The energy per synaptic event is defined as the energy divided by the total number of transmitted spikes *N*_tspikes_. *N*_tspikes_ is composed of all occurring spikes times the number of outgoing connections from the respective sending neurons.

On SpiNNaker, a maximum number of neurons to be simulated on each core can be specified in the current software implementation. Populations of neurons are specified in the PyNN script, and a core runs a subset of the neurons from at most a single population; neurons from several populations are not combined. Thus if the network specifies a population of 100 neurons and a second population of 50 neurons and requests 90 neurons on a core, three cores will be used split as 90 on the first core, 10 on the second core and 50 on the third, despite the fact that the last 10 neurons of the first population could be combined with the second population within the given constraints. This is purely due to software engineering decisions; it is easier to keep track of the neurons if a core can only contain part of a single population. This could change in a future version of the software.

SpiNNaker boards are either single boards or combined in units of 3 boards. This makes it easier to deal with the coordinate space on the boards. The boards are physically placed into subracks of 24 boards, where each subrack has a backplane providing power to the boards and a 48-port switch providing networking from the outside world to the boards, with one 100 Mb/s Ethernet connection to each board and a second Ethernet connection to the management processor on each board. This external network is used purely for I/O interactions with the boards; network traffic generated during the simulation is passed entirely via the SpiNNaker network on and between the boards. The management processors allow each of the boards to be powered on independently and the links between boards to be turned on and off; thus within a single 24-board rack, boards are allocated either individually or in groups of units of 3 boards. The software is capable of working out an approximate number of boards required for the simulation and then requesting this allocation; in the case of the cortical microcircuit model simulation, 6 boards are requested.

Once a SpiNNaker machine has been allocated, it interacts with a host computer, which reads the machine configuration information (including the layout of the machine and any hardware issues such as faulty cores, chips and links), and works out how the neural network is to be run on the machine. Once this has been determined, the network data is generated and loaded, and the network is run.

For estimates of power consumption we connected a single 24-board rack to a consumer power measurement device at the mains socket, and ensured that there were no other users; thus only 6 of the 24 boards were ever active, with the other 18 remaining switched off. The power measurement device integrates the power usage over time providing an energy consumption in kWh at chosen points in time. An estimate of baseline power results from a measurement with the power on but with all the boards powered off. This allows eliminating the power usage of the rack itself, including the power consumption of the network switch, though not the cooling system, which is activated dynamically. A webcam pointing at the meter takes snapshots of the device at appropriate moments in the simulation setup, loading, execution, and result extraction phases to obtain readings for these stages. These measurements yield the total energy consumption for each execution phase in steps of Δ*E* = 0.01 kWh. When computing the power for a phase of duration *T*, we propagate this measurement inaccuracy according to Δ*P* = 1/*T*·Δ*E*. The software of SpiNNaker presently does not allow turning off individual cores and chips. Therefore it is not possible to subtract the power consumption of unused hardware components on each board.

For both NEST and SpiNNaker, the respective machines are exclusively used for the simulations under consideration. There are no contributions to the overall energy consumption from other jobs running.

## 3. Results

### 3.1. Conceptual separation of biological time and wall-clock time

Model neurons in the SpiNNaker system are updated at regular intervals in wall-clock time; this allows the simulation to be divided between several CPUs with independent timers, and still maintain reasonable synchronization across the system. A typical setting in previous studies is *h*_w_ = 1 ms. As SpiNNaker was originally designed for real-time operation, the interpretation of the biological model of the time span between two update events was considered to be identical to the wall-clock time passing between two updates, *h*_b_ = *h*_w_. However, from the point of view of a general simulation engine the two quantities are conceptually not identical. If the equations of a neuron model require updates in intervals of 0.1 ms in order to achieve the desired numerical accuracy, *h*_b_ can be interpreted as *h*_b_ = 0.1 ms. Without further changes to the parameters of the SpiNNaker system this means that the dynamics of the neural network now evolves 10 times slower than wall-clock time. However, the number of spikes occurring per second of wall-clock time is now reduced by a factor of 10: if a neuron model emits a spike within 1 ms with probability 1, the probability to emit a spike within an interval of 0.1 ms is 0.1. Therefore, if the limiting factor for reliable operation of the hardware is the number of spikes per second of wall-clock time, it might be possible to increase the clock-speed of the system by a factor of 10 (*h*_w_ = 0.1 ms) and recover real-time performance while safely staying within the limits of the communication bandwidth.

However, the communication bandwidth is rarely the limiting factor in simulations on SpiNNaker; we must also consider the CPU cycles required to process each spike received, and each synapse the spike activates. The design specifications of SpiNNaker assume a connectivity of 1, 000 incoming synapses per neuron; the cortical microcircuit model has a value closer to 10, 000, which means that the simulation must be slowed down further to accommodate the extra computation this requires, otherwise the synchronization of the simulation is liable to drift between the cores, and the results will be unpredictable and unreliable. If we assume that at the design specifications of SpiNNaker, the computation is split roughly as 10% or 20, 000 CPU cycles per time step for neural updates and 90% or 180, 000 CPU cycles for synapse processing, setting the time step to 0.1 ms means that 10 times more work is required for neural processing, giving 200, 000 CPU cycles per ms of biological time but the amount of work for synapses remains constant at 180, 000 CPU cycles per ms as the number of synaptic events per time step is reduced by a factor of 10. Setting the number of synapses per neuron to 10, 000 means 10 times more work, or 1, 800, 000 CPU cycles per ms, leading to a total of 2, 000, 000 CPU cycles per ms of biological time for all the computation required. This can be achieved by slowing down the simulation by a factor of 10. In practice, there are additional overheads in these processes, and we achieve reliable operation when *h*_w_ = 2 ms, meaning a slow-down of the dynamics compared to real time by a factor of 20.

### 3.2. Steps toward implementation on SpiNNaker

We iteratively refined the SpiNNaker interface for PyNN to extend the range of functions covered, and to match their syntax and functionality. Furthermore, we enabled running long simulations, where it was previously only possible to have short runs due to the memory filling up with the recorded data. We also implemented the NEST connectivity routine used by Potjans and Diesmann (2014) on SpiNNaker. The representation of multapses (multiple synapses between a pair of neurons; Crook et al., 2012) was already supported in the software, and all that was required was to generate the connectivity data using the host Python software. The large number of synapses, however, were more of an issue; for previous models, the synaptic data for the entire network was generated in advance of execution. The representation of this data in Python required a large amount of RAM on the host PC. We therefore modified the software to perform initial estimates of resource usage on the SpiNNaker machine based on statistical information about the PyNN connectors, including the multapse connector created specifically for this network. The software now generates the actual connectivity data lazily for each core, one by one, just prior to loading onto the machine, reducing the RAM usage on the PC by orders of magnitude. This method is also faster, reducing the data generation time from more than 8 h to around 1 h. This process could be parallelized to further reduce the data generation time, but this is not done in the current software due to restrictions of Python running in parallel.

The limited resources and efficiently implemented data structures within the SpiNNaker simulation environment enforce a limit of 1.6 ms for connection delays when the biological time step is 0.1 ms, due to the use of 16-element ring buffers for synaptic inputs (for an explanation of ring buffers in neural network simulations, see for instance Morrison et al., 2005). The mean delay of excitatory connections in the microcircuit model is 1.5 ms, so it was not initially possible to draw delays from a normal distribution with reasonable width. To resolve this issue, we implemented a “delay extension” mechanism, whereby delays >1.6 ms were split into a multiple of 1.6 ms steps plus a remainder:

$$\begin{array}{l} {\text{ delay}}_{\text{extended}}=\left\lfloor \frac{{\text{delay}}_{\text{total}}}{1.6\text{ ms}}\right\rfloor \times {\text{delay}}_{\text{total}},\\ {\text{delay}}_{\text{remaining}}\;={\text{ delay}}_{\text{total}}-{\text{delay}}_{\text{extended}}. \end{array}$$

The extended delay is handled by a separate core. Knowing that the delay is a multiple of 1.6 ms allows up to 8 such multiples, or 12.8 ms of delay, to be simulated within the limited resources of this core. These can in principle be chained together allowing any delay, but a single additional core combined with the maximum 1.6 ms in the neuron model itself (a total of 14.4 ms) was deemed sufficient in this model. Besides enabling longer delays to be represented, support for distributed delays was added.

The synaptic weights of the model are of the order of 10^2^ pA. On SpiNNaker, a single synapse is represented by a 32-bit number consisting of 8 bits for the target neuron index (allowing up to 256 neurons per core—the identity of the core is not stored in the synaptic word but in the routing tables, so neurons can have more than 256 targets), 4 bits for the delay (allowing up to 16 values, as described above), 2 bits for the synapse type (excitatory or inhibitory), and 16 bits for the synaptic weights; 2 bits are reserved to allow for increasing the number of synapse types. The 16-bit weight values are stored as fixed-point values, but the position of the binary point is adjusted to ensure that also the largest summed synaptic inputs occurring in the simulation can be represented. The reason for this adjustment is that when a spike is received on a core, the weight from each synapse is added into one of the 16-delay ring-buffers, each of which is also 16 bits in size; thus ideally the combination of the additions of several weights should not overflow the buffer. Additionally, an appropriate degree of precision is required to represent the weight values given; for example, using 8 bits for the decimal part of the numbers would lead to a precision of 1/(2^8^) nA≈4 pA. This would give a fairly large error as a fraction of the synaptic weights in our simulations. The calculation of the position of the binary point is done by finding the maximum value likely to be added to any single ring buffer element. In previous implementations, this was done by simply adding together all the weights incumbent on each of the neurons and taking the maximum. This guaranteed that the ring buffer elements would never overflow but tended not to leave enough precision in the weights for correct representation, especially not in the case of the cortical microcircuit where there are a large number of connections, but a relatively low firing rate. This calculation was therefore updated to combine the statistics of the connectivity to get an approximate upper bound on the sum of the weights in any ring buffer element. This is done by firstly assuming an average input spike rate and choosing a scale factor σ to use for the overhead in the calculation. We treat the ring buffer elements equally, since, although the delays are distributed, the ring buffer element that represents the given delay from the current time step is moving as the time steps progress. Also the number of ring buffer elements is unimportant, since, regardless of how many there are, delay values will appear which could place the weight in any one of the elements. Thus, the calculation concerns any delay ring buffer element. We then look to the distribution of the weights combined with the timing of the spikes, since it is the arrival of a spike that causes a weight to be added to the ring buffer. For the purpose of determining the maximal resolution of the synaptic weights that allows the summed inputs to the neurons to be represented, we assume a Poisson distribution in the number of spikes arriving. This does not mean that inputs in the model indeed need to be Poissonian; the estimated resolution will work under moderate deviations from Poisson statistics, and the resolution can be decreased in case of highly synchronous input. Under the Poisson assumption, we can expect this same distribution in the addition of the weights to the ring buffer elements. Taking the mean and standard deviation of the weights, we can then compute an expected mean and standard deviation in the sum of the weights in any ring buffer element. Thus, we can approximate the maximum weight as a number of standard deviations above this mean value. Applying these assumptions, the following closed-form solution is derived from standard results on means and variances of products of independent variables:

(1) $$\begin{array}{l} v_{r}=n\;w_{\text{mean}}^{2} \end{array}$$

$$\begin{array}{l} U=\text{round}\left[n+3\sqrt{n}\right]\\ v_{w}=\frac{e^{-n}n\;w_{\text{var}}\left(-n^{U}+e^{n}\Gamma_{\text{inc}}\left[1+U,n\right]\right)}{\Gamma \left[1+U\right]},\\ M=n\;w_{\text{mean}}+\sigma \sqrt{v_{r}+v_{w}} \end{array}$$

where *M* is the expected maximum value over time in any of the delay ring buffer elements, which is calculated using *n*, the average number of expected incoming spikes in a time step; *w*_mean_, the mean of the incoming weights; σ, the number of standard deviations above the mean for safety overhead (set to 5 here); *w*_var_, the variance of the incoming weights; the gamma function Γ, and the incomplete gamma function Γ_inc_. In the cortical microcircuit simulation, we take the expected rates within the network to be 30 spikes/s and use the known rates of the Poisson generators for calculating the synaptic weight resolution. Requiring that *M* from Equation (1) can be represented leads to weights with 6 or 7 bits for the integer part (respectively allowing summed input values with integer parts up to 2^6^−1 = 63 nA and 2^7^−1 = 127 nA) and 10 or 9 bits respectively for the fractional part, depending on the total number of incoming synapses to the population in question, since it is the summed weights in the ring buffer elements that determine the necessary resolution. In terms of weights of single synapses, with 10 bits for the fractional part of the number, the weight of 0.0878 nA would be represented as 0.0869140625, and with 9 bits for the fractional part, the representation is 0.0859375. By comparison, the nearest double-precision (64-bit) floating point representation of 0.0878 is 0.08780000000000000304201108747292892076075077056884765625, and the nearest single-precision (32-bit) representation is 0.087800003588199615478515625; using half-precision floating point (16-bit) for the value would result in the value 0.08779296875 being used. Thus, the precision of the weight values on SpiNNaker is reasonable given the 16 bits available for use. During the single-neuron tests, the whole 16 bits were used as the fractional part of the number, leading to 0.0878 being represented as 0.087799072265625. Note that even with this calculation, the chances of overflow of the buffer are non-zero, and overflows will still likely occur in long-running simulations. Thus the software counts the number of times an overflow occurs and reports this to the user at the end of the simulation.

The communication network of SpiNNaker can support up to 6 million spike packets per second, but it does not cope well with all the traffic occurring within a short time window within the time step. This is exacerbated by the initial synchronization of the simulation engine at the code level, making all cores likely to send spikes at the same time. The neuron cores pause the processing of neurons and thus the sending of spikes whilst they are processing incoming spikes, so after the initial spikes there is some spreading of the network traffic over the time step occurring naturally. However, the Poisson noise generating cores and the delay extension cores have little to no inputs, so they have no automatic spreading of the sending of the spikes over the time step due to time spent processing incoming spikes. Furthermore, in the microcircuit simulations, the spikes tended to be concentrated within a small window within the time step despite the desynchronization due to the processing of inputs. Without any correction, it is likely that all the network traffic will therefore occur within a small window at the start of the time step, and all cores will send simultaneously. To overcome this issue, each core is firstly given a random wait time at the start of every time step. This gives a basic offset so that the first network packet sent by a core is unlikely to be synchronized with that of the other cores. Given the maximum number of network packets to be sent by the application within a single time step, it is then possible to work out an expected minimum number of CPU clock cycles between sending packets within a time step; in the case of neurons each neuron can only send at most one spike per time step, but with Poisson sources, the likely maximum number of spikes per time step has to be calculated statistically. For example, with a 0.1 ms biological time step in real time, a 200 MHz CPU and 100 neurons being executed on the core, there are at most 100 packets to be sent each time step, and 20, 000 clock cycles in which to send them, so there should be 200 clock cycles between the sending of packets. In practice, we spread the packets over half the time step, to allow the spikes to be processed at the receiving end, so there would be 100 clock cycles between packets in this example. If the execution arrives at a point at which a packet is to be sent, but the expected number of CPU cycles since the start of the time step has not passed, the core is simply made to wait until this occurs. On neuron cores, the neurons continue processing spikes during this pause, whereas the delay extension and Poisson generator cores have little else to do when this occurs. Prior to this change, there were quite a few dropped packets in the simulation. With this change no packets were lost during the simulation.

The independent Poisson input sources of the cortical microcircuit model also required modifications of the SpiNNaker software stack. The software was designed for an input spike rate to each neuron around 10 spikes/s and assuming each source neuron to have synapses onto multiple target neurons within each population. This means that there is not too much network traffic, and that each Direct Memory Access (DMA) performed when a spike is received retrieves multiple synapses from the SDRAM, increasing the overall efficiency of the transfer by reducing the overheads of each transfer. The one-to-one connectivity of the Poisson sources coupled with their high firing rate breaks both these assumptions. The revised software contains a heuristic in the placement algorithm which attempts to place one-to-one connected populations on the same chip where possible. This reduces the communication overhead, since only the internal network-on-chip is used to transfer the spikes between the Poisson sources and the populations they feed. Furthermore, the synaptic connectivity data for the one-to-one connected populations are now stored in local Data Tightly Coupled Memory (DTCM); a DMA to transfer the data is no longer required. The high input rates also mean that multiple spikes often need to be sent in a single time step, for which support was added.

As SpiNNaker has limited SDRAM and no other backing store, the storage of recorded data can become an issue, even for short simulations. The improved software overcomes the problem by calculating the maximum duration of the simulation before the SDRAM is filled by the recorded data. The simulation runs for this period, pauses whilst the data are extracted from the machine, and then resumes. This repeats until the simulation has covered the required duration.

### 3.3. Comparison of single-neuron results between NEST and SpiNNaker

**Figure 2:**
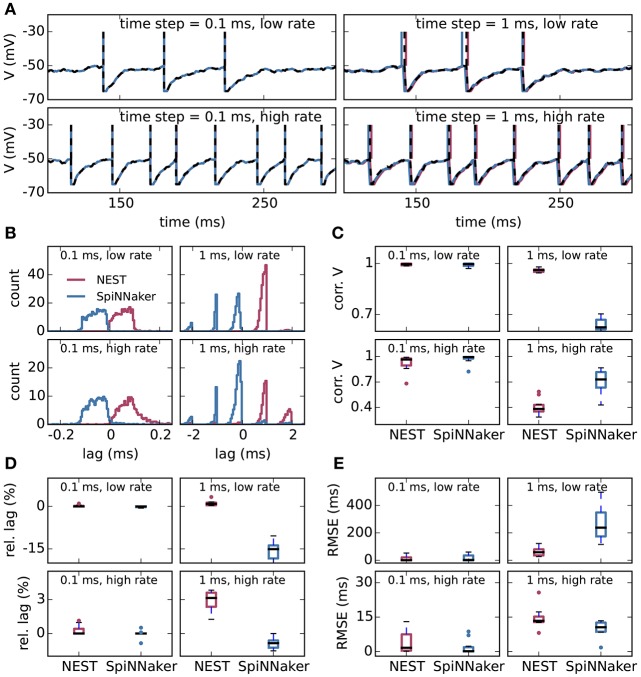
Single-neuron tests for SpiNNaker and grid-based NEST simulations. **(A)** Example membrane potential traces of leaky integrate-and-fire model neurons (parameters as in Table 1) receiving Poisson input with a “low rate” of 8, 000 spikes/s and a “high rate” of 10, 000 spikes/s (rows) for computation time steps 0.1 ms and 1 ms (columns). Red, NEST with spikes constrained to the grid; blue, SpiNNaker; black dashed curves, NEST with precise spike timing. Spike times are indicated by vertical lines. For time step 0.1 ms, all subthreshold traces overlap nearly precisely. **(B)** Average cross-correlation histograms over 10 simulations between binned spike trains from grid-based NEST (red) and SpiNNaker (blue) and those from NEST with precise spike timing. **(C)** Pearson correlation coefficients between membrane potential traces. **(D)** Accumulated fractional lead or lag in spike times. **(E)** Root mean square error of spike timing after correcting for accumulated lead or lag. All comparisons are with NEST with precise spike timing. **(C–E)** Thick black lines, median across 10 repeat simulations; boxes, interquartile range (IQR); whiskers extend to the most extreme observations within 1.5 × IQR beyond the IQR.

**Figure 3:**
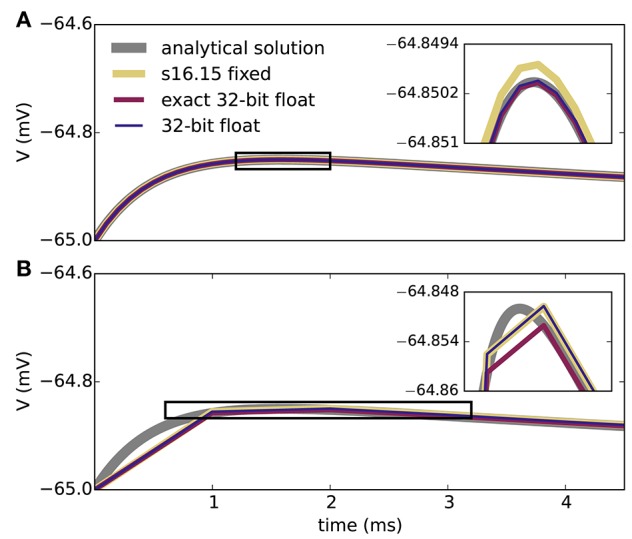
Membrane potential excursion for different computation step sizes and numerical representations. Panels show the peak region of the response of the model neuron on the SpiNNaker system to a single input spike (neuron parameters as in Table 1) for computation time steps 0.1 ms **(A)** and 1.0 ms **(B)**. The ISO specification s16.15 fixed represents signed fixed-point numbers with 16 bits for the integer part and 15 bits for the fractional part; 32-bit float uses a single-precision ISO standard floating-point representation. Both s16.15 fixed and 32-bit float separate out the integration of the exponential decay of the synapses and the LIF neuron model (exponential integration), though each component uses a closed-form solution. Exact 32-bit float uses a single closed-form solution that encompasses both the exponential decay of the synapses and the LIF neuron model. This exact integration corresponds precisely to the analytical solution sampled at the integration time step. The s16.15 format leads to the same synaptic weights as with the 16-bit format in Figure 2. Double-precision (64-bit) floating-point numbers give membrane potential excursions that are visually indistinguishable from the single-precision results. Insets enlarge the membrane potential traces delineated by the black boxes.

Figure 2 shows the results of the single-neuron tests, comparing the simulation output of grid-based NEST and SpiNNaker with that of NEST with precise spike timing, which provides a near-exact reference solution (cf. section 2.1). The example membrane potential traces in Figure 2A show that both simulators achieve a high accuracy. SpiNNaker displays a slight lead and grid-based NEST a slight lag with respect to the precise solution that is visible especially at 1 ms resolution. These deviations are also apparent in the cross-correlation histograms of the binned spike trains (Figure 2B; for a comparison of different numerical solvers, see Rotter and Diesmann, 1999). The histograms for time step 1 ms contain multiple peaks due to the 1, 000 Hz rhythm imposed by the grid-constrained input spikes. The lag of grid-based NEST is due to the fact that spike times are always rounded up to the nearest grid point, not down (Morrison et al., 2008; Krishnan et al., 2017). The early spiking of SpiNNaker is likely to be due to the use of fixed-point numerical representations and the separation of the exponential decay of the synaptic inputs from the integration of the membrane equation, as shown for a single input spike in Figure 3. Since the fixed-point synaptic weights in these simulations are slightly smaller than the floating-point values, the increased postsynaptic response with the fixed-point representation must be due to the limited resolution of the other neuron parameters and variables. The separated integration leads to consistently higher values of the membrane voltage in response to the incoming spike, but for 0.1 ms time steps, the numeric type appears more influential than this separation (insets of Figure 3). The deviations for the lower-rate simulations are slightly smaller than those for the high-rate simulations, because the limited memory of the dynamics causes subthreshold traces with different initial conditions to converge under identical inputs. In terms of membrane potential correlations, both simulators perform well at 0.1 ms resolution (Figure 2C). At 1 ms resolution and low rates, NEST outperforms SpiNNaker in terms of membrane potential correlations, accumulated spike lead or lag, and spike time precision (Figures 2C–E). This may be explained by the greater contribution of subthreshold dynamics, which NEST integrates exactly, as compared to spiking dynamics at low rates. At 1 ms resolution and high rates, SpiNNaker outperforms NEST on all three measures (Figures 2C–E).

### 3.4. Comparison of network results between NEST and SpiNNaker

**Figure 4:**
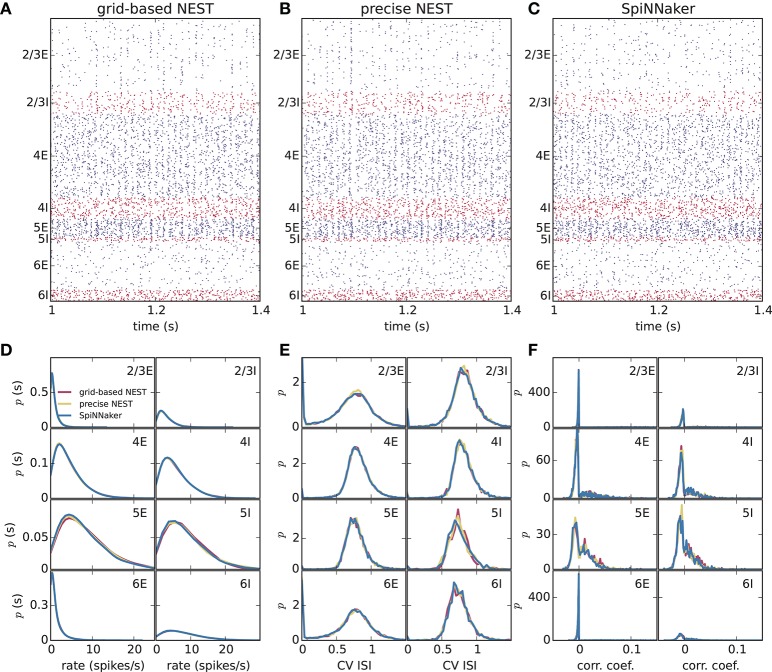
Spiking output of the cortical microcircuit model with DC input. **(A–C)** Raster plots showing spike times (dots) of excitatory neurons in blue and of inhibitory neurons in red. The spikes of 5% of all neurons (vertical) are displayed. **(D–F)** Distributions of spiking activity for each of the eight populations of the model. **(D)** Single-neuron firing rates of all neurons averaged over the last 9 s of the simulation. **(E)** CV ISI, a measure of irregularity of all neurons. **(F)** Correlation coefficients between binned spike trains for 200 neurons in each population. Histogram bin widths are determined by the Freedman-Diaconis rule.

**Figure 5:**
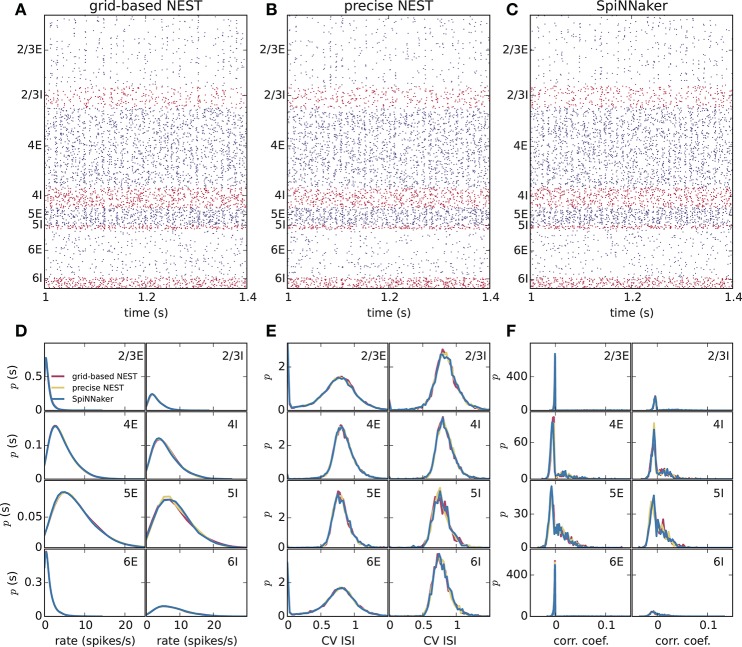
Spiking output of the cortical microcircuit model with Poisson input. Same display and parameters as in Figure 4.

Different slowdown factors were tested on SpiNNaker to determine the minimal slowdown factor at which no spike loss occurs in the simulation of the cortical microcircuit model. Based on the biological time step of 0.1 ms compared to the 1 ms design specification, this slowdown has to be at least a factor of 10. As explained in section 3.1, additional slowdown is necessary to enable processing the high input rates to the neurons. At a slowdown factor of 20 with respect to real time SpiNNaker simulates this model without any spike loss. Also, the chosen precision for the synaptic weights prevents any overflows of the synaptic ring buffers from occurring in the SpiNNaker simulations. Therefore, differences between the NEST and SpiNNaker simulation results are only caused by floating-point vs. fixed-point numerical representations, exact subthreshold integration vs. separate integration of membrane voltage and synaptic inputs, and different random number generator seeds. Grid-based NEST, NEST with precise spike timing, and SpiNNaker produce closely similar spiking statistics, both for DC input (Figure 4) and for Poisson input (Figure 5). The raster plots of the spiking activity (Figures 4A–C, 5A–C), a standard tool for the visual inspection of multi-channel spike data (Grün and Rotter, 2010), bring out the similarity. Despite the different initial conditions and different realizations of the connectivity, and also different input realizations in the case of Poisson input, distributions of average single-neuron firing rates (Figures 4D, 5D), spiking irregularity (Figures 4E, 5E), and correlation coefficients between binned spike trains (Figures 4F, 5F) match closely between the three simulation methods and for all neural populations.

**Figure 6:**
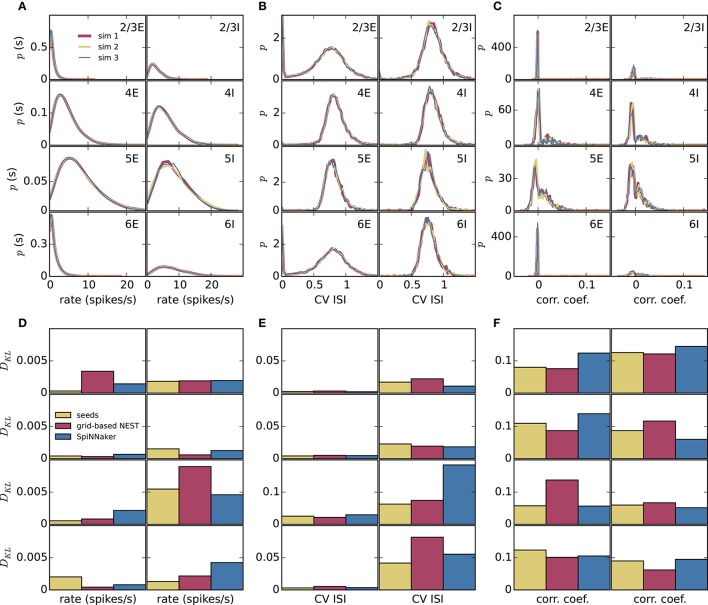
Comparison of influence of random number generator seeds and simulation method. **(A–C)** Distributions of dynamical properties of the microcircuit model for each of the 8 neural populations obtained using NEST with precise spike timing. Results for three simulations, each of 10 s duration discarding a 1 s transient, with different random seeds. **(D–F)** Kullback-Leibler (KL) divergences from NEST with precise spike timing as a reference, using results from NEST with precise spike timing and different random seeds (mean of KL divergences for two simulations with different seeds), and results from grid-based NEST and SpiNNaker. **(A,D)** Time-averaged single-neuron firing rates. **(B,E)** Coefficient of variation of interspike intervals. **(C,F)** Pairwise correlations between binned spike trains.

To assess how meaningful the differences between the simulation methods are, we compare these differences with those caused by the random number generator seeds alone. We perform three simulations of the microcircuit model with Poisson input for 10 s with NEST with precise spike timing with different random seeds for the connectivity, initial membrane potential distributions, and Poisson generators (Figure 6). In each case, we compare the distributions of rates, CV ISIs, and correlations, discarding a 1 s transient as before, in terms of the Kullback-Leibler divergence between the smoothed histograms. Since the simulations with the three methods (grid-based NEST, NEST with precise spike timing, and SpiNNaker) each use different random seeds, differences between the simulation results for these methods include the influence of the seeds, particularly in view of the finite length of the data. The results shown in Figures 6D–F indicate that the influence of the random seeds is comparable in size to the combined influence of the simulation method and the seeds. Thus, the simulation method itself contributes little to the variation in the dynamical properties of the microcircuit model, indicating in particular that SpiNNaker's fixed-point numerics and approximations in the subthreshold integration do not compromise accuracy for networks of the given type.

### 3.5. Performance

**Figure 7:**
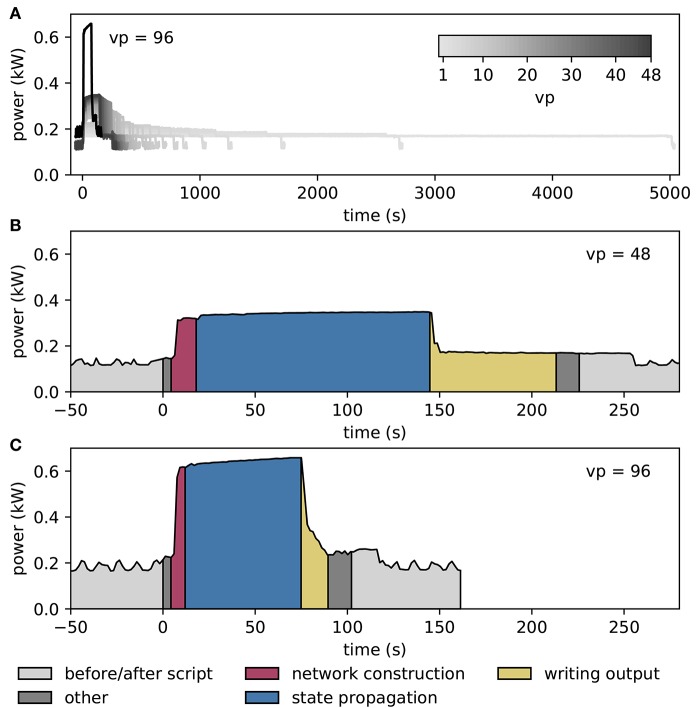
Temporally resolved power consumption during microcircuit simulation with NEST on HPC cluster. **(A)** Total power consumption as a function of time of a single compute node using 1–48 threads (gray code) and of two compute nodes with 48 threads per node (*vp* = 96, black). The curves terminate with the end of the simulations (for 10 s of biological time in all cases). **(B)** Power consumption in labeled execution phases of a simulation (legend) on a single compute node with 48 threads. **(C)** Execution phases for two compute nodes with 48 threads per node.

Figure 7 shows results from measurements of the power consumption during benchmark simulations with NEST on one and two compute nodes of an HPC cluster. The simulations use an increasing number of threads on the single node, and all threads supported by the hardware on the two nodes (Figure 7A). The measured power consumption rises during script execution and we observe that it increases with the number of *vp*s whereas the required time decreases. In Figures 7B,C, we enlarge the traces for *vp* = 48 and *vp* = 96, respectively, and indicate the execution phases of the script. Prior to the execution of the script, the system exhibits a fluctuating baseline power consumption of the switched-on nodes; the baseline is higher for two nodes compared to one node. The phases “network construction” (red) and “state propagation” (blue) are the main phases as they refer to the setup of neurons and connections and the propagation of the dynamical state of the neural network, respectively. The color-coded areas for these phases have approximately the same size, indicating a similar energy consumption. The “writing output” phase transfers spike times from the simulation engine to file buffers after the dynamics has reached its final state. The corresponding PyNN function gathers data from all processes and uses only one thread per node for writing. Time spent otherwise during script execution is denoted in dark gray. These intervals correspond for instance to loading Python modules and setting simulation parameters before the network construction starts, and plotting the spiking activity after writing output. When the script has terminated, the timestamps are written to file, and after that, the power consumption returns to the baseline level.

**Figure 8:**
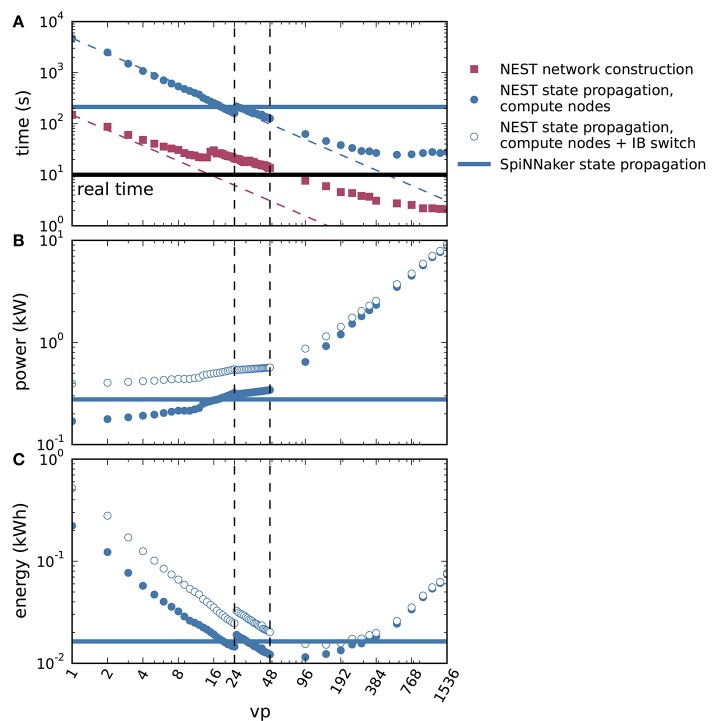
Time to solution and energy consumption of NEST and SpiNNaker simulations. **(A)** Duration of network construction (red square markers) and propagation of the dynamics (blue circular markers) vs. number of virtual processes for NEST simulations. Dashed lines represent ideal scaling. Black horizontal line indicates stretch of biological time simulated (10 s). **(B)** Mean power consumption during propagation for NEST simulations measured at involved compute nodes (filled markers) and with an additional power offset for the InfiniBand (IB) switch (open markers). **(C)** Energy consumption during propagation for NEST simulations measured at involved compute nodes (filled markers) and with an additional power offset for the IB switch (open markers). On a single node, *vp*s bind initially to cores on one processor (up to *vp* = 12), then to cores on the second processor (up to *vp* = 24, left vertical dashed line), and finally to the second hardware thread on each core (up to *vp* = 48, right vertical dashed line). Blue horizontal lines in each panel indicate duration, power and energy of state propagation, respectively, for a SpiNNaker simulation. All panels in double-logarithmic representation.

We further spread the NEST simulations across up to all 32 compute nodes of the HPC cluster with 48 threads on each node (*vp* = 1, 536), shown in Figure 8. Figure 8A demonstrates the parallel scalability of network construction and propagation of the dynamics by showing the measured times together with the ideal linear expectations. The propagation time saturates at about three times the biological time. The jump in propagation time after *vp* = 24 coincides with the onset of hyperthreading. Network construction time continues to decrease over the full range of compute nodes but exhibits an intermediate increase starting at *vp* = 15 (see Ippen et al., 2017 for a general discussion of network construction time). Figure 8B shows the power consumption averaged over the propagation phase as a total across all nodes used in the particular simulation. The change in slope at *vp* = 48 is due to the successive switching on of additional nodes. Integrating the power consumption traces over the propagation interval yields the energy consumption as depicted in Figure 8C. Due to the decrease in propagation time and the concomitant growth in power consumption with increasing *vp*, the energy consumption reaches a minimum at *vp* = 96, i.e., at two nodes. Thus, the hardware configuration requiring the minimal energy-to-solution is neither the one with the smallest number of hardware components involved nor the one with the shortest time-to-solution, but a system of intermediate size. The energy per synaptic event for the optimal configuration is 4.4 μJ.

Apart from the compute nodes, however, we also have to take other components of the cluster into account to estimate the total energy-to-solution. The HPC cluster requires two service nodes, with an estimated combined contribution of approximately 300 W, comparable to the base level of two compute nodes. Two Ethernet switches and one InfiniBand (IB) switch consume, based on their data sheets, a maximum 64 and 226 W, respectively. During the propagation phase, only the compute nodes and the IB switch are required. Figures 8B,C assess how an additional power offset accounting for the IB switch affects the power and the energy consumption as functions of *vp*. The increase in power consumption is crucial for small *vp*, but it is almost irrelevant for simulations across multiple compute nodes (large *vp*). We also observe that the minimum energy to solution shifts to a larger *vp*, and conclude that simulations become more efficient if distributed across more hardware. Including the contribution of the IB switch, the minimal energy per synaptic event is obtained at *vp* = 144 and equals 5.8μJ. At this number of virtual processes, the simulation takes about 4.6 times real time.

**Figure 9:**
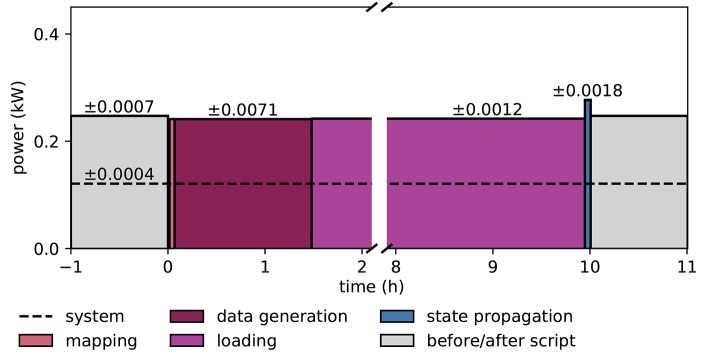
Power consumption during microcircuit simulation with the SpiNNaker system. The vertical axis shows the average power consumption during the color-coded execution phases. The power (black solid horizontal line segments) is computed from measurements of the energy consumption (colored areas) of the execution phases. The dashed horizontal line indicates the baseline measurement with the rack powered on but all 6 boards switched off. Numbers above black lines state the error in power estimation. Before and after the execution of the simulation script (light gray segments) boards are switched on and booted but idle. Power consumption during mapping (salmon) is set identical to consumption in data generation phase (raspberry). Propagation of the dynamical state by 10 s of biological time takes 200 s (blue segment). The respective power estimate results from the propagation by a longer stretch of biological time (1, 000 s) for increased accuracy.

Figure 9 illustrates the power consumption of the SpiNNaker system, derived from the measurements of the energy consumption for each execution phase (see section 2.5). The background power usage caused by the network switch, the active cooling systems, and the power supply itself explains half of the total power consumption. As in the case of the HPC cluster we do not include any cooling of the room into the measure. The mapping phase is where the software of the host computer reads the machine configuration and then uses this description to work out which parts of the neural network are to be executed on which chip, and the routes taken by network traffic that is to traverse the machine during simulation. Power consumption is mostly the same as in the idle phase, since the machine is only briefly contacted during this phase, with the rest of the work being done on the host computer. The data generation phase creates the data for each core; this includes the neuron parameters and synaptic matrices, as well as other SpiNNaker-specific data. Again, the machine is not in use during this phase, and hence could be turned off. The loading phase transfers the data generated on the host computer to the SpiNNaker machine. Although this requires communication with the machine, power consumption is still low, because only two cores on the machine are active at any time during this phase. These are the monitor core on an Ethernet-connected chip and the monitor core on the chip storing the data in memory. During the phase of state propagation, the power consumption increases significantly above the level of the idle state, reflecting the work done by the cores. The energy per synaptic event consumed during the propagation phase is 5.9 μJ.

Duration, power, and energy consumption of the propagation phase are included as horizontal lines in Figure 8 to facilitate a comparison with NEST. The energy consumed by the SpiNNaker simulation is close to the minimum energy of the NEST simulation for compute nodes and the IB switch, leading to a similar result for the energy per synaptic event on both systems.

Previous measurements of the SpiNNaker system indicated the approximate power usage of each chip to be 1 W when fully loaded, and the overhead for each board itself, excluding the chips, to be approximately 12 W. The 6 boards and 217 chips used in the present study thus predict a power consumption of (6 × 12+217) W = 289 W. This is close to the 277 W measured during the state propagation phase, indicating that a calculation based on the number of chips and boards in use delivers a good estimate of the power consumption during this phase. With 48 chips per board, there are 48 × 6−217 = 71 unused chips, of which the power consumption is measured along with that of the active chips, but not taken into account in the back-of-the-envelope calculation. The fact that this calculation already gives a higher value than the measurement suggests that the power consumption of the unused chips is negligible.

## 4. Discussion

On the example of a full-scale cortical microcircuit model (Potjans and Diesmann, 2014), the present work demonstrates the usability of SpiNNaker for large-scale neural network simulations with short neurobiological time scales and compares its performance in terms of accuracy, runtime, and power consumption with that of the simulation software NEST. With ~0.3 billion synapses, the model is the largest simulated on SpiNNaker to date, as enabled by the parallel use of multiple boards. The result constitutes a breakthrough: as the model already represents about half of the synapses impinging on the neurons, any larger cortical model will have only a limited increase in the number of synapses per neuron and can therefore be simulated by adding hardware resources. The synaptic time constants and delays of the model necessitate a shorter integration time step (here, 0.1 ms) than the original 1 ms design specification of SpiNNaker. The higher resolution is achieved by a conceptual separation of biological time and wall-clock time. For the microcircuit model the current software stack of SpiNNaker requires the number of neurons per core to be set to exactly 80. This restriction is the result of the number of routing entries available on each router in the machine, combined with the current algorithm for assigning keys to the neurons. As a consequence of the combination of required computation step size and large numbers of inputs, the simulation has to be slowed down compared to real time. In future, we will investigate the possibility of adding support for real-time performance with 0.1 ms time steps. Reducing the number of neurons to be processed on each core, which we presently cannot set to fewer than 80, may contribute to faster simulation. More advanced software concepts using a synapse-centric approach (see Knight and Furber, 2016) open a new route for future work.

We assess accuracy by comparing grid-based NEST and SpiNNaker simulations with NEST simulations with precise spike timing, which provide a highly accurate reference solution. For the cortical microcircuit model, we consider firing rates, coefficients of variation of interspike interval distributions, and cross-correlations between binned spike trains. Which measures to use to quantify accuracy and which level of accuracy is considered to be acceptable has to be determined on an individual model basis from the acceptable range of desired model behaviors. For instance, for the microcircuit model, it is important that the simulations preserve asynchrony and irregularity of spiking, and differences in firing rates between the neural populations. Although models and their desired outcomes are diverse, these measures are also chosen because they characterize several fundamental aspects of single-neuron and population activity, and are therefore relevant to a wide range of models. Despite its fixed-point arithmetic, SpiNNaker is able to achieve comparable accuracy on these measures to that of NEST. Conversely, NEST executed on a high-performance cluster achieves speed and power efficiency comparable to the performance of SpiNNaker for some settings and in addition enabling a flexible trade-off between runtime and energy-to-solution. These results take into account that runtime and energy consumption should be assessed while controlling for simulation accuracy. For networks even larger than the cortical circuit considered, where runtime becomes strongly communication-dominated on traditional architectures, the asynchronous update of SpiNNaker may yet give it an advantage in terms of efficiency outside the scope of the present study due to an ability to simply expand the number of cores used by the simulation with minimal communication overhead. Thus, larger networks can be simulated in a weak scaling scenario where network size increases without increasing the rates of neuron state updates and synaptic events per neuron. For networks of any size, SpiNNaker is expected to yield accurate results as long as the simulation speed is chosen such that no spikes are lost and the resolution of the synaptic weights is sufficiently high. Further work is required to assess its scaling of runtime, memory, and energy consumption with network size.

The cortical microcircuit model consists of leaky integrate-and-fire (LIF) model neurons. To assess accuracy in a more controlled setting, we also consider single-neuron simulations. This reveals that grid-based NEST and SpiNNaker have similarly high accuracy at 0.1 ms time steps, with NEST slightly lagging behind and SpiNNaker slightly leading the precise solutions. This respective lag and lead can be attributed to details of the neuron and synapse implementation, with NEST using exact integration (Rotter and Diesmann, 1999) for the subthreshold dynamics, whereas SpiNNaker uses fixed-point representations and a separation of the integration of the synaptic exponential decay and the neuron model. In terms of single-neuron dynamics, NEST performs relatively better at low spike rates, whereas SpiNNaker performs relatively better at high spike rates. The high accuracy of NEST for low rates may be due to the greater contribution of subthreshold dynamics in this condition.

Previous work has provided estimates of the power consumption of SpiNNaker executing spiking neural network models. Stromatias et al. (2013) instrument a 48-chip SpiNNaker circuit board to measure power consumption directly. They model locally and randomly connected networks of up to 200, 000 Izhikevich model neurons and up to 250, 000 LIF model neurons with a 1 ms time step and over a billion synaptic events per second, with a total board power consumption in the region of 30 W, arriving at a total energy per synaptic event of around 20 nJ. Subtracting baseline power, the incremental energy per synaptic event is found to be 8 nJ. Sharp et al. (2012) describe a small but detailed cortical model running with a 1 ms time step on a 4-chip SpiNNaker board instrumented to measure power. The model has 10, 000 neurons and 4 million synapses, consuming just under 2 W, and the energy breakdown yields an incremental cost of 100 nJ per neuron per ms and 43 nJ per synaptic event, with a total energy per synaptic event of 110 nJ.

The present work measures the power consumption of the microcircuit model simulations on SpiNNaker and uses strong scaling with NEST on a high-performance compute cluster. At the optimal setting for NEST, with 144 virtual processes, the energy consumption of the compute nodes per synaptic event is 5.8 μJ, and for SpiNNaker the equivalent measurement is 5.9 μJ. There are several factors that contribute to the lower efficiency of SpiNNaker when running this model compared to the earlier studies, which mostly relate to the model being distributed sparsely over the SpiNNaker hardware, thereby causing baseline power to be amortized across many fewer synaptic events. The principal factors are: the use of a 0.1 ms time step, rather than the standard 1 ms; the biologically realistic number of ~10,000 synapses per neuron compared with the ~1,000 typical in neuromorphic models; and the highly distributed sparse connectivity of the biological model. With further software optimizations we expect such a network, with 80,000 neurons and 0.3 billion synapses, to map onto around 320 SpiNNaker cores—about half of a 48-chip board instead of the 6 boards used here—and to run in real time. With a 30 W power budget for half a board and 10 billion synaptic events over 10 s this yields 30 nJ per synaptic event, in line with the earlier total energy figures and two orders of magnitude below the present value. This ratio highlights the potential and the importance of further improvements of the software stack of the SpiNNaker system; this could include the use of the synapse-centric approach (Knight and Furber, 2016), which has been shown to accommodate the 0.1 ms time step and high synapse count better than the current mapping, but this is not yet available within the SpiNNaker tool flow. This would hopefully enable real-time operation of SpiNNaker during the network propagation phase, as well as reduce the number of cores and thus boards required for this simulation, and so result in a reduction in the power per synaptic event.

Mammalian brains consume about 6 kCal/day = 0.3 W per 1 billion neurons, of which roughly half is consumed by the cerebral cortex (Herculano-Houzel, 2011), and a substantial fraction is due to action potential signaling (Attwell and Laughlin, 2001; Lennie, 2003). In the human brain, with its 10^11^ neurons (Herculano-Houzel, 2012), cortex makes up close to 20% in terms of the number of neurons (Pakkenberg and Gundersen, 1997), so that we obtain 0.15 W per 2 × 10^8^ cortical neurons. Assuming 10^4^ synapses per neuron and an average spike rate of 4 spikes/s (Attwell and Laughlin, 2001), we arrive at an energy consumption of 0.15 W/(2 × 10^8^×10^4^×4 spikes/s) = 19 fJ per synaptic event. Since commonly used extracellular recording methods may miss a large fraction of neurons that are silent or nearly so, the average spike rate of cortex may actually be lower (Shoham et al., 2006). Taking an estimate of 0.1 spikes/s based on whole-cell recordings (Margrie et al., 2002; Brecht et al., 2003), we obtain 0.15 W/(2 × 10^8^×10^4^×0.1 spikes/s) = 760 fJ per synaptic event. These estimates indicate that with our computing systems and for the given model we are between about 7 and 9 orders of magnitude removed from the efficiency of mammalian cortex.

It is important that all contributing components are taken into account when comparing computing systems (noted by Hasler and Marr, 2013). In the present study, we have excluded the energy required for controlling the room temperature. In addition, we have ignored the contributions of the host computer and the Ethernet network to the energy consumption of the SpiNNaker simulations, and for the NEST simulations we have excluded the energy consumed by storage units, service nodes, and Ethernet switches in the derivation of the energy per synaptic event. The reason for ignoring these components is that they are in principle not needed during the phase in which the dynamics is propagated, except for data output, of which the contribution depends on the goal of the study. Similarly, it is in principle possible to power off the unused cores on the SpiNNaker boards, so the power usage of these cores could also be discounted. However, comparison of our measured power consumption, which includes both active and unused cores, with estimates based on active cores only, suggests that the contribution of the unused cores is negligible. Since we measured the power consumption of entire nodes on the HPC cluster, the measurements include cores not used for the NEST simulation up to the point where full nodes are assigned. This contribution is limited by the cores controlling their clock speed in steps depending on computational load, but could be discounted altogether.

Currently, both simulation engines require initial simulations to find the optimal setup in the first place, and these should be taken into account when evaluating their total energy consumption. In future, runtime models may be developed to estimate the optimal setup for a given network. This way, no additional simulations would be needed in order to determine at least a reasonable parallelization.

While the power consumption measurements described here only concern the phase in which the dynamics is propagated, on SpiNNaker the time taken to generate and load the network architecture is much longer, and needs to be addressed by future work. The present implementation already substantially reduces the time it takes to generate the connectivity. Work in progress includes developing the ability to generate the connectivity, which makes up the bulk of the data used by the simulation, on the cores of SpiNNaker. This, as in the case of NEST (Morrison et al., 2005), has the potential to further reduce the network generation time through parallelization, as well as speeding up the loading of data by only transferring the parameters for the statistical generation of the synapses rather than the instantiated connections as is done now.

The current work considers networks of point neurons with static current-based synapses. In general, neural network models can contain more complex features, such as multi-compartment neuron models, conductance-based effects, and plasticity. Since such features increase the time required for neuron and synapse processing, they reduce the maximal rate at which the neurons on the SpiNNaker hardware can receive inputs and the number of neurons that can be mapped to a core while maintaining simulation speed. For instance, depending on the exact model and parameters chosen, simple pairwise spike-timing-dependent plasticity with additive weight dependence reduces both these quantities by a factor of 7 on SpiNNaker with the current software stack, and a factor of 2.5 with synapse-centric mapping of the network to the cores (Knight and Furber, 2016). More complex synaptic plasticity models with multiple dynamical variables like those described by Benna and Fusi (2016) can also be implemented but would further lower the number of neurons per core and their maximal input rates for a given simulation speed. One trend in computational neuroscience is toward ever larger-scale complex models (e.g., Traub et al., 2000; Lundqvist et al., 2006; Yu et al., 2013; Markram et al., 2015; Schmidt et al., 2016). Also such models can in principle be implemented on SpiNNaker; however, the scaling of the required resources and the corresponding simulation performance remain to be investigated.

Our comparison of SpiNNaker and NEST highlights concepts like accuracy, the influence of randomness, concreteness of use cases, and a common formal model specification that need to be considered when comparing systems of this sort. The concepts herein discussed facilitate the evaluation of other low-power platforms such as TrueNorth (Akopyan et al., 2015) and ROLLS (Qiao et al., 2015), and those that are similar to SpiNNaker but with other architectural features, such as described by Moradi et al. (2018).

Porting network models to dedicated hardware is a useful exercise to help identify requirements (the right product is built) and benchmark the results against existing simulation software (the product is built right). This gives us confidence that the co-design process in which we are engaged in the framework of a sequence of large-scale European consortia will continue to successfully guide us in the future. Close collaboration between hardware developers and computational neuroscientists ensures that the product can be used for realistic applications by its intended user community.

## Author contributions

SvA, AR, and JS wrote the simulation code. SvA, JS, AR, and MH wrote the analysis and plotting scripts. SvA and JS performed the NEST simulations. JS did the HPC performance measurements. AR and AS developed the SpiNNaker support software. AR performed the SpiNNaker simulations. SvA, AR, JS, MH, MS, and MD analyzed the data. AR and MH were supervised by DL and SF. JS and MS were supervised by MD. All authors jointly did the conceptual work and wrote the paper.

### Conflict of interest statement

The authors declare that the research was conducted in the absence of any commercial or financial relationships that could be construed as a potential conflict of interest.
